# Klebsiella variicola Reference Strain F2R9 (ATCC BAA-830) Genome Sequence

**DOI:** 10.1128/MRA.00329-21

**Published:** 2021-07-01

**Authors:** Ulises Garza-Ramos, Nadia Rodriguez-Medina, Luis Lozano-Aguirre, Jesús Silva-Sanchez, Margarita Sanchez-Arias, Jeannet Rodriguez-Olguin, Esperanza Martínez-Romero

**Affiliations:** a Instituto Nacional de Salud Pública (INSP), Centro de Investigación Sobre Enfermedades Infecciosas (CISEI), Cuernavaca, Morelos, Mexico; b Centro de Ciencias Genómicas, Laboratorio de Genómica Evolutiva, Universidad Nacional Autónoma de México, Cuernavaca, Morelos, Mexico; c Centro de Ciencias Genómicas, Universidad Nacional Autónoma de México, Cuernavaca, Morelos, Mexico; University of Arizona

## Abstract

Klebsiella variicola F2R9 was isolated from banana root, and its sequence has been deposited as ATCC BAA-830. It corresponds to sequence type 11 (ST11) and KL16 and contains no identifiable plasmids. The genome showed few antimicrobial resistance and virulence genes and several plant association genes. The strain showed susceptibility to most antimicrobials and avirulent behavior.

## ANNOUNCEMENT

Klebsiella variicola was described as a new bacterial species in 2004 and is considered to belong to the Klebsiella pneumoniae complex (1). Studies on the molecular epidemiology of *K. variicola* (2) and comparative genomics of multidrug-resistant isolates (3) as well as species-differentiating approaches within the K. pneumoniae complex have been reported. *K. variicola* may cause different infections, in some cases with a high mortality, and is currently considered an emerging pathogen of humans (4).

Klebsiella variicola F2R9 was deposited as ATCC BAA-830 and is also described as CFNE 2004 and DSM 15968. A single colony of F2R9 was grown on LB agar and then inoculated into LB broth at 37°C with shaking at 200 rpm overnight. Genomic DNA was extracted with the MasterPure complete DNA kit (MC85200; Epicentre). Nanopore and Illumina sequencing libraries were prepared using rapid barcoding sequencing (SQK-RBK004; Oxford Nanopore Technologies) and the NEBNext Ultra DNA library prep kit (E7370L; New England BioLabs), respectively. MinION sequencing was conducted with an R9 12-type flow cell (FLO-MIN106D) and the MinKNOW software with base calling. A total of 2 × 12,146,103 paired-end reads (75 bp long) were sequenced with the Illumina platform and 23,446 reads (average length, 11,476 bp) were sequenced with the Nanopore platform. Illumina reads were quality verified by FastQC software v0.11.8 (https://www.bioinformatics.babraham.ac.uk/projects/fastqc/) and trimmed with Trim_Galore v0.6.4 (https://www.bioinformatics.babraham.ac.uk/projects/trim_galore/). Both forward and reverse reads were trimmed (15 bp eliminated) from the 5′ end to avoid poor quality or bias. *De novo* hybrid assembly of *K. variicola* F2R9 with long MinION and short Illumina reads was performed using Unicycler v0.4.7 (5). The genome assembly of *K. variicola* F2R9 contains one contig that represents a circularized chromosome (5,519,584 bp) with no identifiable plasmids.

The chromosome was annotated with the NCBI Prokaryotic Genome Annotation Pipeline (6), which identified 5,125 genes and 122 RNA genes, including 25 rRNA genes (9 5S, 8 16S, and 8 23S), 83 tRNA genes, and 14 noncoding RNA (ncRNA) genes. The full genome was analyzed for predicted antibiotic resistance genes using ResFinder 4.1 (7). ResFinder identified LEN-17, *oqxA*, *oqxB*, *fosA*, and *fosA7*. The predicted virulence factor genes identified were *entABCDEF*, *kfuABC*, *mrKABCDFHIJ*, *fimABCDEFGHIK*, *kva*, *kvb*, *kvc*, *kvd* and *kvg* (8), *uge*, *ureABCDEFG*, and *wabG*.

The sequence type (ST) that corresponds to *K. variicola* F2R9 is ST11 according to the *K. variicola* MLST database (https://mlstkv.insp.mx) (2) and the capsular serotype KL16 according to the Kaptive database (9). F2R9 only showed ampicillin resistance in culture medium, with avirulent behavior in mice (10). We recommend using the Klebsiella variicola F2R9 (ATCC BAA-830) genome as the reference genome mainly for *K. variicola* comparative genomics studies and for *in vitro* and *in vivo* assays.

### Data availability.

This whole-genome shotgun project has been deposited in DDBJ/ENA/GenBank under the accession no. CP072130. The raw sequence reads are available under the SRA accession no. SRR14145449 and SRR14530140.
